# Effect of 10-Valent Pneumococcal Vaccine on Pneumonia among Children, Brazil

**DOI:** 10.3201/eid1904.121198

**Published:** 2013-04

**Authors:** Eliane Terezinha Afonso, Ruth Minamisava, Ana Luiza Bierrenbach, Juan Jose Cortez Escalante, Airlane Pereira Alencar, Carla Magda Domingues, Otaliba Libanio Morais-Neto, Cristiana Maria Toscano, Ana Lucia Andrade

**Affiliations:** Federal University of Goiás, Goiania, Goiás, Brazil (E.T. Afonso, R. Minamisava, A.L. Bierrenbach, O.L. Morais-Neto, C.M. Toscano, A.L. Andrade);; Pontifical Catholic University of Goiás, Goiania (E.T. Afonso);; Ministry of Health, Brasilia, Brazil (J.J.C. Escalante, C.M. Domingues);; University of Brasília, Brasília (C.M. Domingues);; University of São Paulo, São Paulo, Brazil (A.P. Alencar)

**Keywords:** Pneumonia, pneumococcal vaccines, PCV10, time-series analysis, vaccination, hospitalization, bacteria, infant, Brazil

## Abstract

Pneumonia is most problematic for children in developing countries. In 2010, Brazil introduced a 10-valent pneumococcal conjugate vaccine (PCV10) to its National Immunization Program. To assess the vaccine’s effectiveness for preventing pneumonia, we analyzed rates of hospitalization among children 2–24 months of age who had pneumonia from all causes from January 2005 through August 2011. We used data from the National Hospitalization Information System to conduct an interrupted time-series analysis for 5 cities in Brazil that had good data quality and high PCV10 vaccination coverage. Of the 197,975 hospitalizations analyzed, 30% were for pneumonia. Significant declines in hospitalizations for pneumonia were noted in Belo Horizonte (28.7%), Curitiba (23.3%), and Recife (27.4%) but not in São Paulo and Porto Alegre. However, in the latter 2 cities, vaccination coverage was less than that in the former 3. Overall, 1 year after introduction of PCV10, hospitalizations of children for pneumonia were reduced.

*Streptococcus pneumoniae* infections are the leading cause of bacterial pneumonia, meningitis, and sepsis among children (*1*,*2*); in developing countries, these infections account for almost a half million deaths among children <5 years of age (*3*). In Brazil, the largest country in South America, the role of *S. pneumoniae* in pneumonia in children is considerable (*4*,*5*).

Brazil is composed of 5 administrative regions with different climatic and socioeconomic characteristics. In 2010, the estimated population of infants (children <12 months of age) was ≈2,800,000, and the infant mortality rate was 17 deaths per 1,000 live births (*6*,*7*). In Brazil, the main reason for hospitalization of infants is pneumonia (*6*).

Vaccination with pneumococcal conjugate vaccine (PCV) is a public health intervention to prevent pneumococcal disease. PCV has been in use since 2000, when a 7-valent vaccine (PCV7) was licensed in the United States for routine use in children. In 2010, PCV7 was replaced by a 13-valent vaccine. Recently, a 10-valent pneumococcal conjugate vaccine (PCV10) was licensed in Brazil; this vaccine includes the same serotypes that are in PCV7 (4, 6B, 9V, 14, 18C, 19F, 23F), plus 3 more (1, 5, and 7F) (*8*).

In 2010, Brazil introduced PCV10 into its routine National Immunization Program. Previously, no PCV had been incorporated into the routine immunizations. The vaccination was introduced in all cities from March through September 2010; 3 doses (at 2, 4, and 6 months of age) plus 1 booster (at 12–15 months of age) were recommended. Two routine catch-up schedules were also in place: 1) two doses for children 7–11 months of age plus a booster at 12–15 months of age, and 2) one dose for children 12–24 months of age. PCV10 is not given to children >24 months of age (*9*).

In Brazil, vaccination of children with PCV10 is free through the National Unified Health System (*10*). By October 2011, the mean vaccination coverage rapidly reached 80% for a full primary series for children <12 months of age in >5,000 municipalities (Brazilian Ministry of Health, unpub. data).

Studies that assessed the effect of PCV7 found a statistically significant reduction in the overall incidence of invasive pneumococcal disease and hospitalizations for pneumonia among children <2 years of age shortly after the first year of vaccination (*11*–*14*). Our aim was to assess the effectiveness of PCV10 for reducing hospitalizations for all-cause pneumonia. We analyzed trends in rates of hospitalization for pneumonia among children soon after the introduction of PCV10 in Brazil. Ethical approval was granted by the Ethics Committee, Federal University of Goiás, Goiania, Brazil. 

## Methods

### Data Sources

We conducted an interrupted time-series analysis by using individual-level secondary data from the Hospitalization Information System of the National Unified Health System from January 2005 through August 2011. The Hospitalization Information System records ≈75% of all hospitalizations in Brazil and 60%–80% of the hospitalizations for the cities in the analyses (*15*). During the study period, there were no major changes in the amount of hospital care provided by the National Unified Health System.

Variables in the Hospitalization Information System are demographics, date of admission/discharge, residential address, hospital code, and International Classification of Diseases 10th Revision (ICD-10) codes for primary and secondary diagnoses. Because the Hospitalization Information System database is mainly used for reimbursement purposes, the likelihood that hospitalizations would be underreported and that data would be missing are small (*16*).

The structure of the Hospitalization Information System made it possible for 1 episode of hospitalization for a given patient to be recorded multiple times. Additional records might be generated when patients remain hospitalized longer than anticipated. To avoid including duplicate records, we used a deterministic record linkage algorithm to find records for the same patient (*17*). We then considered that consecutive records of the same patient with a 14-day interval between discharge and reentry belonged to the same episode of disease (*18*).

**Figure 1:**
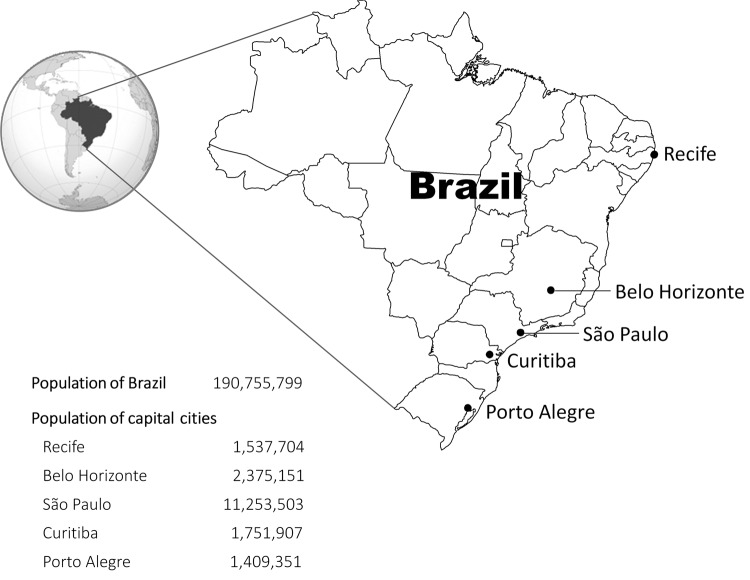
Capital cities of Brazilian states, and their populations, in which effectiveness of 10-valent pneumococcal vaccine was studied. Population data obtained from Brazilian Census 2010.

We studied 5 state capital cities in Brazil: Belo Horizonte, Curitiba, Recife, São Paulo, and Porto Alegre (Figure 1). The cities were initially selected from a list of 10 cities participating in an ongoing case–control study evaluating the effect of PCV10 vaccination on pneumococcal disease. The selection of cities for the case–control study was based on data quality and willingness of the local surveillance teams to participate in the study. Of the initial 10 cities, 5 were excluded a priori from the time-series analysis; 3 cities had not reached vaccination coverage of at least 75% for the first dose of vaccine (primary series) 3 months after vaccine introduction, and 2 cities were excluded because of poor data quality in the initial descriptive analyses. The 5 chosen cities account for 50% of the population of the state capitals of the country and are located in 3 of the 5 administrative regions of the country.

The annual numbers of live births were obtained from the Live Birth Information System and used to calculate the annual population of children 2–24 months of age. The monthly population was calculated by interpolating an exponential growth model to the annual data.

### Definitions

We identified Hospitalization Information System records of children 2–24 months of age who were hospitalized from January 2005 through July 2011 with specific ICD-10 codes: pneumonia (J12-J18), bronchiolitis (J21), respiratory causes (J00–J99), nonrespiratory causes, and all causes (*19*). We considered nosocomial pneumonia more likely to be reported as a secondary discharge diagnosis; therefore, only the primary diagnosis of the first record of each episode of disease was used in all data analyses.

In a descriptive analysis, which included only the prevaccination period, we obtained average annual numbers and rates of pneumonia hospitalizations for each city and the proportion of pneumonia out of all respiratory causes and out of all causes of hospitalizations. Specific pneumococcal pneumonia–coded cases in the Hospitalization Information System represented only 0.06% of the pneumonia cases reported in the system because confirming bacteriologic pneumonia in children is difficult; thus, we considered use of pneumococcal pneumonia–coded cases not appropriate in a time-series analysis.

### Vaccination Coverage

In Brazil, the National Immunization Program, established in 1973, led to high rates of coverage (*20*). PCV10 vaccination was introduced in March 2010 in all selected cities except Porto Alegre, where it started in June 2010. PCV10 coverage data for each city were obtained from the National Immunization Program vaccine coverage database of the National Unified Health System, in which number of vaccine doses and administrative vaccination coverage are made available for all municipalities in the country. Numerator data are obtained from the number of doses administered in the vaccination rooms, by vaccine type, patient age, and municipality. PCV10 coverage for a full PCV10 primary series (3 doses) was estimated as the number of third doses of PCV10 administered (numerator) to children <12 months of age divided by the number of births in a population over time in each municipality (denominator) multiplied by 100 (see Technical Appendix, for sources of data on vaccination coverage).

**Figure 2:**
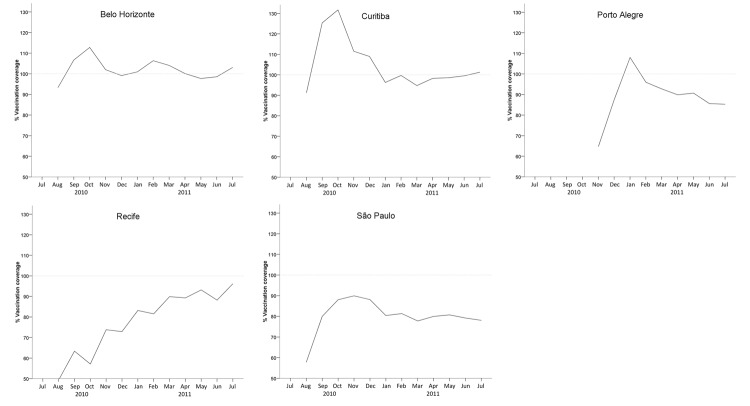
Monthly coverage for third dose of 10-valent pneumococcal vaccine achieved 11–14 months after vaccination among children <12 months of age in 5 cities in Brazil. Dotted horizontal lines represent 100% vaccination coverage.

We calculated the moving average of vaccine coverage for every 3-month period. The value attributed to a given month was the average vaccine coverage in that month and the coverage for the months before and after the given month. This calculation was done to smooth out short-term vaccination coverage fluctuations (Figure 2).

### Data Analyses

In the interrupted time-series analysis, 3 immunization periods were defined: prevaccination, transition, and postvaccination. The prevaccination period was January 2005–February 2010 and had 62 time points (monthly data) in the final model (except for Porto Alegre, which had 65 time points because vaccination introduction was delayed for 3 months). The transition period was the time of vaccine introduction through 4 months after. The postvaccination period was the time after the transition period; it comprised 14 time points in the analysis (3 fewer for Porto Alegre). The transition period was excluded from analysis, although it is shown in the figures. The time-series analysis was based on a generalized linear model for rates of hospitalizations for pneumonia and for nonrespiratory causes by using the negative binomial distribution with a logarithmic link function and an offset equal to the log of the population divided by 100,000 (*21*). Residual analyses showed no substantial deviations from model assumptions. The main outcome was rates of hospitalization for all-cause pneumonia. The explanatory variables in the model were calendar month (to control for seasonality), linear trend over time (to control for preexisting trends), and a variable equal to 1 after vaccination and 0 otherwise. After estimation of the models, 2 outputs were presented: 1) the percentage change in hospitalization rates, which compare the prevaccination and postvaccination periods and their corresponding p values and 95% CIs, and 2) a graph showing the predicted hospitalization rates for pneumonia and their 95% CIs for the postvaccination period based on models fitted with data for the prevaccination period. With the latter output, it is possible to visually evaluate the observed and the predicted monthly hospitalization rates for the postvaccination period; that is, the rates that would have resulted had the changes during the transition period not taken place.

Sensitivity analyses for each city compared models with and without removal of the months of July and August 2009. This comparison was an attempt to reduce potential bias resulting from the influenza epidemic. To compare the estimated percentage change for hospitalizations for pneumonia and nonrespiratory causes, we performed separate time-series analyses. In theory, the vaccination-induced percentage change would be higher for hospitalizations for pneumonia than for nonrespiratory causes, although a minority of possible pneumococcal disease codes was included in nonrespiratory causes (such as meningitis). The percentage change for hospitalizations for nonrespiratory causes was expected to reflect influences other than the PCV10 vaccination effect that might have concomitantly affected the data series. We expected the effect of reporting/processing delays to still be present during the postvaccination period, therefore inducing an artificially lower number of hospitalizations in more recent months (although we had discarded the most recent ones from analysis). To compensate for this effect, we calculated the differences between the percentage changes in rates of hospitalizations for pneumonia and nonrespiratory causes for each city. The equality of the percentage changes for hospitalization for pneumonia and nonrespiratory causes was tested by using the Wald test (*22*).

The observed trends for bronchiolitis, respiratory, and all-cause hospitalizations are shown for comparison. The linkage/classification procedures were conducted by using STATA version 12.0 (www.stata.com/), and the statistical analysis was done by using R (www.r-project.org/).

## Results

In the 5 cities, 197,975 hospitalizations of children 2 months to 2 years of age were identified during the study period; 109,155 (55.1%) were for respiratory causes, including 59,636 (30.1%) for pneumonia. During the prevaccination period, the rates of pneumonia hospitalizations varied substantially by city (Table 1).

**Table 1:** Rates of hospitalization for pneumonia among children 2 months–2 years of age, Brazil, prevaccination period, 2005–2009*

City	No. cases, annual mean (± SD)	Rates, annual mean (± SD)	% Hospitalizations for pneumonia/hospitalizations for all respiratory causes	% Hospitalizations for pneumonia/hospitalizations for all causes
Belo Horizonte	939 (133)	1.643 (217)	53.4	34.8
Curitiba	359 (49)	790 (114)	72.2	27.9
Recife	538 (41)	1.304 (107)	47	23.4
São Paulo	3999 (312)	1.247 (103)	61.4	36.1
Porto Alegre	292 (38)	863 (112)	25.8	15

*Pneumonia identified by International Classification of Diseases, 10th Revision, codes: J12–J18.

Figure 2 shows the moving average for PCV10 coverage (percentage of children <12 months of age who received all 3 doses of PCV10). Vaccination coverage varied by city. Belo Horizonte and Curitiba rapidly reached 100% coverage and maintained stable rates of ≈100% from September 2010 on. Recife showed a tendency toward sustained and continuously rising coverage over the study period, eventually reaching ≈100%. Porto Alegre reached 100% coverage on January 2011, followed by a gradual decrease to 85% in July 2011. São Paulo coverage increased to 90% in November 2010, after which it continuously declined, reaching 75% in July 2011.

**Figure 3:**
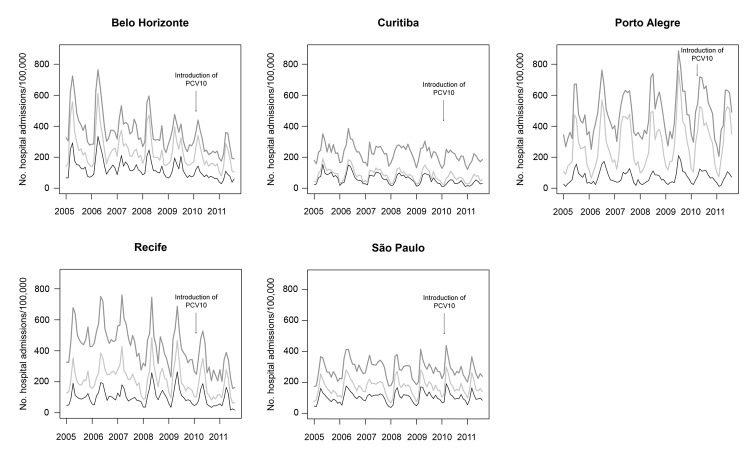
Trends in rates of hospitalization for pneumonia (black) and for all respiratory causes (light gray) and all causes (dark gray) among children 2 months–2 years of age in 5 cities, Brazil, January 2005–August 2011. PCV10, 10-valent pneumococcal vaccine.

Trends in patterns of hospitalization rates for pneumonia, respiratory causes, and all causes are shown for each city (Figure 3). Seasonal variations are evident for all cities. The contribution of pneumonia to the total number of hospitalized patients varied widely by city but not by years. Rates of hospitalization for all causes, particularly from mid-2007 on, decreased notably for Belo Horizonte and Recife; however, the observed reductions in rates of hospitalization for pneumonia for these cities and for Curitiba seem to be restricted to the postvaccination period (from mid-2010 on).

**Figure 4:**
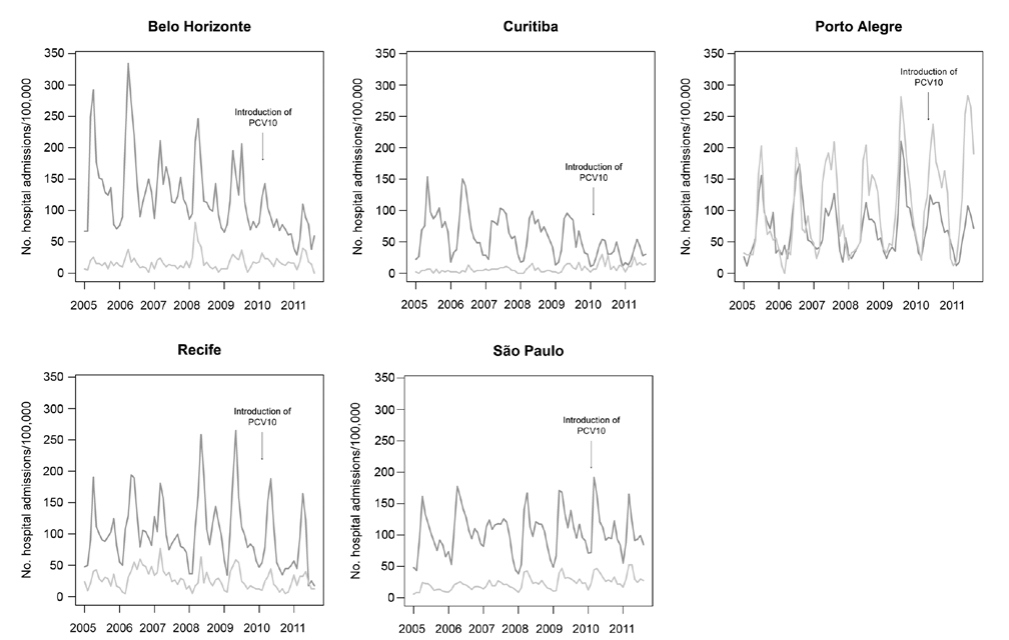
Trends in rates of hospitalization for pneumonia (dark gray) and bronchiolitis (light gray) among children 2 months–2 years of age in 5 cities, Brazil, January 2005–August 2011. PCV10, 10-valent pneumococcal vaccine.

Rates of hospitalization for bronchiolitis were lower than those for pneumonia in all cities except Porto Alegre (Figure 4). The seasonal variations of bronchiolitis and pneumonia were mostly parallel and were found for all cities. Hospitalization rates progressively increased in the more recent years for Porto Alegre, São Paulo, and possibly Curitiba.

**Figure 5:**
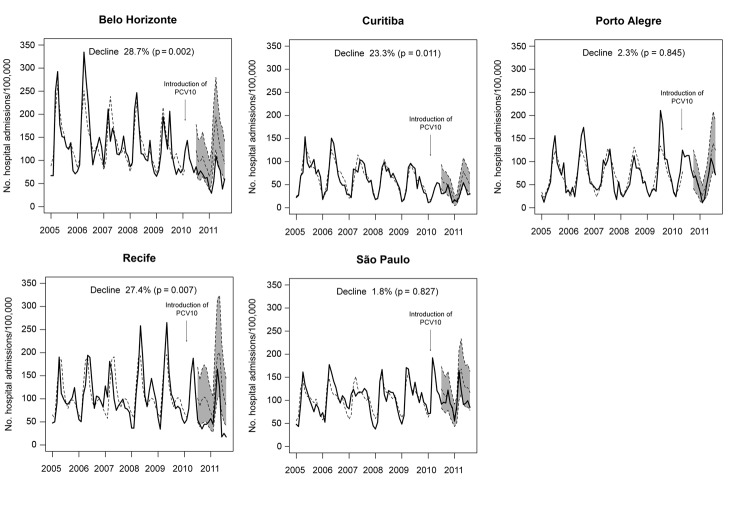
Observed (solid lines) and predicted (dashed lines) rates of hospitalization for pneumonia and 95% CIs (shaded area) among children 2 months–2 years of age in 5 cities, Brazil, January 2005–August 2011. The 95% CIs are shown only for the 4 months after start of vaccination. Decline represents the reduction in hospitalizations for pneumonia. PCV10, 10-valent pneumococcal vaccine.

Table 2 and Figure 5 show results derived from the same time-series models. During the postvaccination period, rates of hospitalization for pneumonia decreased significantly (p<0.001) in Belo Horizonte (−40.3%), Curitiba (−37.6%), and Recife (−49.3%). Rate reductions were borderline significant for São Paulo (−13.4%; p = 0.074) and Porto Alegre (−23.5%; p = 0.052) (Table 2). Rates of hospitalization for nonrespiratory causes also decreased in all cities, albeit at a lower rate. The following differences between the percentage changes in hospitalization rates for pneumonia and nonrespiratory causes represent our best estimate of the vaccination effect: Belo Horizonte (−28.7%), Curitiba (−23.3%), Recife (−27.4%), São Paulo (−1.8%), and Porto Alegre (−2.3%). During the postvaccination period, reductions in rates of hospitalization for pneumonia did not differ significantly from rates of hospitalization for nonrespiratory causes in São Paulo (p = 0.827) and Porto Alegre (p = 0.845).

**Table 2:** Annual percent change (trend) and percentage change in rates of hospitalization among children 2 months–2 years of age, Brazil, postvaccination period, January 2005–August 2011

City	Hospitalizations for pneumonia			Hospitalizations for nonrespiratory causes		Difference in change	p value
	% Change (95% CI)	p value		% Change (95% CI)	p value		
Belo Horizonte	−40.30 (−50.88 to −27.44)	<0.001		−11.61 (−23.48 to 2.10)	0.093	−28.69	0.002
Curitiba	−37.59 (−49.63 to −22.68)	<0.001		−14.27 (−23.94 to −3.38)	0.012	−23.32	0.011
Recife	−49.32 (−61.63 to −33.05)	<0.001		−21.93 (−32.18 to −10.13)	0.001	−27.39	0.007
São Paulo	−13.38 (−26.02 to 1.42)	0.074		−11.60 (−19.31 to −3.15)	0.008	−1.78	0.827
Porto Alegre	−23.51 (−41.60 to 0.18)	0.052		−21.18 (−31.08 to −9.86)	0.001	−2.33	0.845

Figure 5 compares the observed monthly rates of hospitalization for pneumonia with the forecasted values that were modeled with use of data exclusively from the prevaccination period. For Belo Horizonte, Curitiba, and Recife, the observed numbers are close to or below the lower limit of the 95% CI, particularly for the most recent months.

## Discussion

This study indicates that the introduction of PCV10 through the routine immunization program in Brazil has effectively lowered rates of hospitalization for pneumonia among children. Rates of hospitalization for all causes declined in 3 of the 5 cities studied (Belo Horizonte, Curitiba, and Recife). In the other 2 cities (São Paulo and Porto Alegre), these rates did not decline significantly, possibly because vaccination coverage for these 2 cities in 2011 was lower (≈80%) than it was in the other 3 cities (>90%). Another possible reason is that Porto Alegre started its vaccination program 3 months after the other cities, so its postvaccination period was shorter, leaving less time for the vaccination to become effective.

Comparison of our results with those of other studies is not straightforward because, to our knowledge, no comparable studies have been published (e.g., effects of PCV10 on rates of hospitalization for all causes). PCV10 has recently been introduced in some countries in North America and Europe. Preliminary evaluations indicate a reduction of invasive pneumococcal disease. In the province of Quebec, Canada, PCV10 was introduced to the routine immunization schedule 5 years after PCV7 was introduced. Data obtained by a sentinel laboratory surveillance network showed lower incidence of invasive pneumococcal disease among children vaccinated with PCV10 than with PCV7 (35.3 vs. 64.1 cases/100,000 person-years) (*23*). In Finland, results of a recent field trial found a marked decrease in the incidence of invasive pneumococcal disease among children who were vaccinated according to a 3+1 or a 2+1 immunization schedule; vaccine effectiveness reached 100% (95% CI 83%–100%) and 92% (95% CI 58%–100%), respectively, after 2 years (*24*). The Clinical Otitis Media and Pneumonia Study conducted at urban sites in Argentina, Colombia, and Panama showed that the efficacy of PCV10 for reducing community-acquired pneumonia and alveolar consolidation among children was 7.3% and 23.4%, respectively (*25*).

For PCV7, studies have already documented its effect on rates of hospitalization for pneumonia among children (*11*,*12*,*26*–*28*). In the United States, the rates of hospitalization for pneumonia were reduced 39%–52%. However, aside from the use of different vaccines, our study is not directly comparable. Vaccination coverage was generally lower in the United States, increasing from 68% to 83% during the postvaccination period, which was much (4 years) longer. This longer time might have allowed time for herd immunity to protect the nonvaccinated population (*26*,*28*). Also, the illnesses compared in each study were not the same; the United States study evaluated dehydration and diarrhea, whereas our study evaluated all nonrespiratory conditions since the rotavirus vaccine was introduced in 2006 to Brazil.

The introduction of rotavirus vaccination might actually be one of the best explanations for the decreasing trends of all-cause hospitalizations in the target age group during the study period. Another explanation is the rapid increase in coverage of the Family Health Programme. This program reached 85% of Brazilian municipalities in 2010 and greatly reduced deaths and hospitalizations of infants for primary-care sensitive diseases like diarrhea and for lower respiratory tract diseases (*29*–*31*).

Across all 5 cities, we found differences in rates of hospitalization for pneumonia before introduction of PCV10. Marked regional differences had already been documented (*32*). Possible reasons, other than differences in health care provision, are variations in epidemiology, demographics, socioeconomic status, and climate. Health care provisions might play a progressively lesser role in explaining the differences in rates of hospitalization for pneumonia because the results of National Household Sample Surveys show a trend toward equity in access to and use of health care facilities (*31*,*33*).

Several potential limitations of our study should be highlighted. Our data represent only the population served by National Unified Health System in Brazil. The findings observed for the 5 capital cities cannot be considered representative of the entire country. We attempted to include mostly community-acquired cases of pneumonia by restricting our analysis to the primary diagnosis for hospitalization. By doing so, we hypothetically increased the proportion of hospitalizations for pneumococcal pneumonia out of all hospitalizations for pneumonia. Because only a few cases have pneumonia listed as a secondary diagnosis, we missed only a few community-acquired cases of pneumonia by not including it.

Information about the extent of coding errors in Brazil is scarce. None of the information is specific to pneumonia. However, the clinical diagnosis of pneumonia has been shown to have high sensitivity and low specificity for ascertaining pneumococcal pneumonia; thus, any bias resulting from misclassification of ICD-10 would be toward reduction of the observed effect of vaccination (*34*).

Our results could also have been influenced by changes in disease diagnosis and management over time. We observed an increase in hospitalizations for bronchiolitis in the cities of Curitiba, Porto Alegre, and São Paulo. Although this increase might represent a real increase in disease incidence and/or severity, a more likely reason could simply be improvements in the diagnosis of bronchiolitis. Thus, hospitalizations for bronchiolitis, which would otherwise be coded as nonspecific pneumonia or other lower respiratory infections, could be increasingly coded correctly in these locations. Potentially, this reduction in misclassification over time would tend to increase the observed effect of vaccination in these cities, but no vaccination effect was observed in Porto Alegre and São Paulo. We have not identified other major changes in diagnosis and reporting habits, including those motivated by knowledge of PCV introduction or the fact that its effect was being assessed.

The study was conducted at the time of the 2009 influenza pandemic, and an influenza A (H1N1) vaccination campaign was conducted in Brazil as a time-limited intervention. This campaign took place from March through June 2010 and achieved high vaccination coverage among children <2 years of age (Brazilian Ministry of Health, unpub. data). This age group was only slightly affected by the pandemic, as evidenced by the lack of a temporary increase in the rates of hospitalization for pneumonia and rates of hospitalization for influenza (data not shown because numbers were so small). Therefore, we consider it unlikely that the pandemic or its vaccination campaign have biased our results.

Any study that uses a time-series method to determine the early effects of a vaccine can be challenged by fluctuations in vaccination coverage and by the natural lag period between vaccination and protection. Moreover, the limitations of using vaccine coverage estimates derived from secondary data collected for administrative reasons are obvious because of the fact that coverage goes beyond 100% for the initial months after start of a vaccination program (Figure 2). The entry of a new vaccine into the immunization program in Brazil usually attracts infants <6 months of age and infants from areas surrounding the municipality. For both situations, the number of doses administered per month are higher than the number of live births per month.

A major challenge to our analysis was dealing with unavoidable delays for reporting the estimated early effect of PCV10 vaccination. Although we found that the number of hospitalizations for all causes decreased during the most recent months of our series, to run the time-series models we still needed as many data points as possible after the vaccine was introduced. The chosen strategy was to subtract the declines for the nonrespiratory hospitalization rates from the pneumonia rates. By doing so, we accounted for as much of the effect of reporting delays as possible.

In conclusion, our data demonstrate that 1 year after its introduction to Brazil, PCV10 reduced hospitalizations for pneumonia among children in 3 of the 5 cities studied. To ascertain the sustainability of this reduction, prospective analyses covering a longer time after introduction of the vaccination program are needed.

Technical Appendix Sources of data on vaccination coverage.
