# Supplementary material for: Effect of 10-Valent Pneumococcal Vaccine on Pneumonia among Children, Brazil
Source: Emerg Infect Dis. 2013 Apr;19(4):589–97. doi: 10.3201/eid1904.121198 (PMC3647414; doi:10.3201/eid1904.121198)
Supplement: Technical Appendix — Sources of data on vaccination coverage. [file 12-1198-Techapp-s1.pdf]

Article DOI: http://dx.doi.org/10.3201/eid1904.121198

Effect of 10-Valent Pneumococcal Vaccine on Pneumonia among Children, Brazil

Technical Appendix

Link to access the third dose of 10-valent pneumococcal conjugate vaccine (PCV10) for children less than one year of age for the capitals of Porto Alegre, Curitiba, Recife, and Belo Horizonte: http://tabnet.datasus.gov.br/cgi/tabcgi.exe?pni/cnv/DPnibr.def

Link to access the third dose of PCV10 for children less than one year of age for the capital of São Paulo:
http://ww2.prefeitura.sp.gov.br/cgi/tabcgi.exe?secretarias/saude/TABNET/API/API.def

Link to access the population (live births) for Minas Gerais State (Belo Horizonte, code number 310620), Paraná (Curitiba, code number 410690), Pernambuco (Recife, code number 261160), Rio Grande do Sul (Porto Alegre, code number 431490) and São Paulo (São Paulo, code number 355030): http://tabnet.datasus.gov.br/tabdata/sinasc/dados/nov_indice.htm.

Population data for the year 2011 for the 5 municipalities were obtained from the Department of Analysis of Health Situation, Ministry of Health/DATASUS (not yet available online).
